# Open Access to Research Is in the Public Interest

**DOI:** 10.1371/journal.pbio.0050048

**Published:** 2007-02-13

**Authors:** Bevin P Engelward, Richard J Roberts

## Abstract

Bevin Engelward and*PLoS Biology* editorial board member Richard Roberts give their perspective on the importance of public access to science.

With very little fanfare, American science will make a sizeable leap forward in the coming year—if Congress and the National Institutes of Health deliver on their promise for public access to medical research. As scientists—one of us a Nobel researcher in biomedical science and one of us a recently tenured faculty member at the Massachusetts Institute of Technology—we may have much to celebrate for scientists of all generations.

As scientists, scientific literature is our lifeblood, because only by reading our colleagues' work can we know where the cutting edge of knowledge currently lies and hence where our work should be directed.

Yet increasingly, subscriptions to the very journals that we must read are becoming too expensive—often in the thousands of dollars. The availability of the information vital to our research is needlessly restricted by the publishers of the scientific literature, who are mainly large commercial entities for whom maximizing profits is their priority. Fortunately, help is at hand. The big “if” remains whether this will happen.

The National Institutes of Health have championed their wish to maintain all federally funded medical research in a publicly accessible online archive maintained by the National Library of Medicine's PubMed Central. It stands to reason that taxpayer-supported science should be available to all. The Pew Trust recently reported that 93 million Americans searched for medical knowledge on the Web to find the latest information for their health conditions, and more importantly, 58% actually took this information to their doctors. The public interest is clear.

What about the interests of science? We know firsthand that faster, barrier-free access to scientific research findings is equally good if not more compelling for science and scientists—senior and early-career researchers alike. It's good for science because knowledge is cumulative—it advances by building on previous knowledge. Research in a vacuum has little or no value; it is infinitely more valuable when shared and used.

Let's also be plain about the advancement of scientists, not just good science. When barriers to access are swept away, research is used more, and that equates to more citations. Scientific peers acknowledge our work more frequently, and as we all acknowledge, citations are the coin of the realm in all scientific fields. Citations demonstrate the significance of a scientist's research and thus advance our careers as well as our work itself.

For younger scientists, being recognized is critical to our professional successes. Making our work openly available is a means of being recognized and emulated. Senior researchers also should be encouraging their graduate students and postdoctoral colleagues to use open access for career advancement.

Even if the prestigious journals in our fields are not yet open access, young scientists always have opportunities to make our work available in an open archive like PubMed Central or at our universities' online institutional repositories. Academic institutions and the science community are forging new, innovative partnerships to advance science and to promote the best minds by harnessing an open-access environment.

For those of us who have dedicated our lives to science, public access is a two-way street. We can more easily read other scientists' works (which helps our research), and they can read ours (making it far more likely to be cited). Younger researchers, educated and raised in the networked digital environment, are used to moving seamlessly from info source to info source. The scientific research environment should respond to and favor this effective work style.

Knowledge itself is seamless, as ideas spark other ideas, or reject unworkable ones. Through public access to science, at last we will have the advantage of being able to move from primary literature to other data sources—and back. Finally, we will have the opportunity within our grasp to follow a research thread in the ideal way, without artificial barriers, gaps, and tariffs, regardless of the type of material or who owns and curates it—and to instantly make connections. In an age rife with the potential for infectious pandemics, bioterrorism, and toxic environmental calamity, and at a time when we need new ways to cure terrible illnesses, public access is our society's compelling answer to accelerating the best science possible. This advance is much needed, both by researchers working in academic settings and in the private sector. Indeed, we should demand no less. We invite our fellow scientists to join in the demand for open access to biomedical literature.

